# Retrieving a Peripherally Dislodged Coronary Stent: A Last Resort Approach

**DOI:** 10.7759/cureus.78941

**Published:** 2025-02-13

**Authors:** Lavina Chandwani, Saurabh Kumar Singh, Anbhigya Kumar Arya, Anwar Ansari, Devesh Kumar

**Affiliations:** 1 Cardiology, Vardhman Mahavir Medical College and Safdarjung Hospital, New Delhi, IND; 2 Medicine, Atal Bihari Vajpayee Institute of Medical Sciences and Dr. Ram Manohar Lohia Hospital, New Delhi, IND

**Keywords:** arteriotomy, drug-eluting stent (des), high division of brachial artery, pci (percutaneous coronary intervention), stent dislodgement

## Abstract

A middle-aged man in his 50s with a history of percutaneous coronary intervention (PCI) in the left main coronary artery (LMCA) and right coronary artery presented with worsening exertional angina. Invasive coronary angiography revealed a significant diffuse lesion distal to the LMCA stent in the proximal left anterior descending (LAD) artery, prompting a decision to proceed with PCI. During the procedure, the stent detached from the balloon and became lodged at the proximal LMCA stent, resulting in hemodynamic collapse. In an attempt to stabilize the patient, the entire guiding catheter, along with the stent-balloon assembly and coronary wire, was withdrawn through the radial sheath. However, the stent became dislodged and migrated to the right brachial artery. After multiple unsuccessful percutaneous retrieval attempts, surgical removal via brachial arteriotomy was successfully performed.

## Introduction

The use of coronary stents during percutaneous coronary intervention (PCI) has become widespread; however, complications such as stent embolization - where a stent dislodges into the systemic or coronary circulation before deployment - remain serious risks [1]. This can lead to life-threatening consequences, including coronary artery thrombosis, acute myocardial infarction, or even death. If the dislodged stent migrates beyond the coronary arteries, the risk of stroke or peripheral artery obstruction increases.

Several factors contribute to stent dislodgement, including coronary calcification, vessel tortuosity, diffuse disease, and the presence of preexisting stents. Additionally, stent design elements, such as strut thickness and metal composition, play a crucial role. Despite advancements in stent technology, lesion preparation techniques, and the widespread adoption of drug-eluting stents (DESs), the incidence of stent dislodgement remains approximately 1% [2]. Consequently, interventional cardiologists must be well versed in techniques for safely retrieving a dislodged stent.

Here, we present a complex case of complete stent dislodgement during PCI of the left anterior descending (LAD) artery, followed by embolization to the brachial artery. After multiple unsuccessful percutaneous retrieval attempts, a definitive surgical intervention led to successful stent removal.

## Case presentation

A middle-aged gentleman in his 50s, with a history of percutaneous transluminal coronary angioplasty in both the left main coronary artery (LMCA) and right coronary artery (RCA), presented with worsening exertional angina three years after the procedure. He had been on guideline-directed medical therapy, and baseline investigations, including a 12-lead electrocardiogram and a two-dimensional transthoracic echocardiogram, showed no abnormalities. Given his worsening symptoms, coronary angiography was performed, revealing patent stents in both the RCA and LMCA but a significant lesion 15 mm distal to the LMCA stent in the proximal LAD artery.

**Figure 1:**
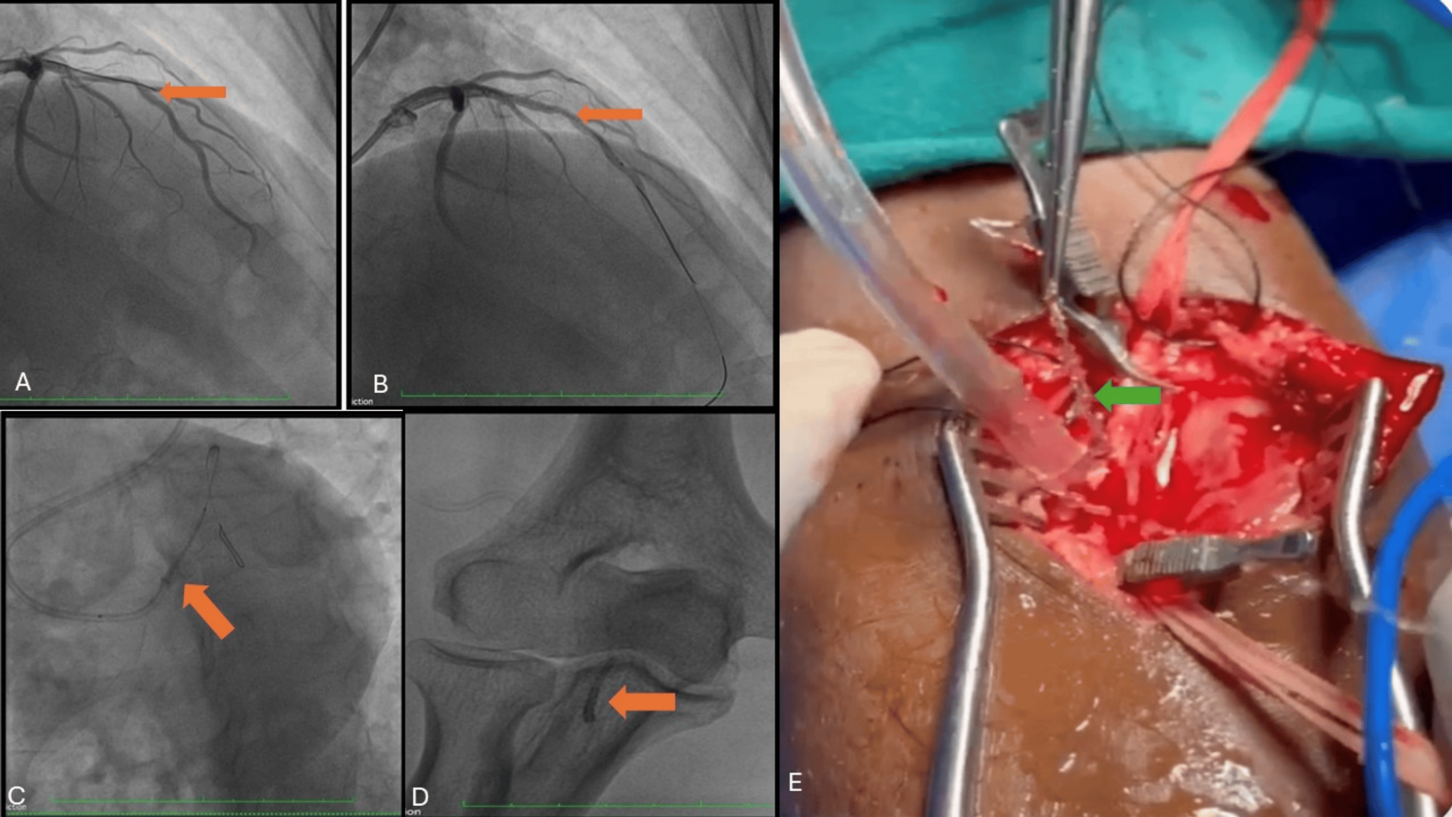
Imaging sequence of stent dislodgement and retrieval (A) Coronary angiogram of the left coronary system in the right anterior oblique cranial view, showing a run-through wire positioned across the lesion (orange arrow). (B) Same view post-balloon dilatation, with the stent deployed across the lesion (orange arrow). (C) Coronary angiogram in the left anterior oblique caudal view, demonstrating stent detachment from the underlying balloon (orange arrow). (D) Fluoroscopic image in the anteroposterior view, showing the dislodged stent at the bifurcation of the right brachial artery (orange arrow). (E) Arteriotomy of the right brachial artery with successful removal of the deformed stent (orange arrow).

Due to the worsening angina, PCI was planned via a 6 French radial approach using a 6 French Judkins Left 3.5 (JL) guiding catheter. The lesion was successfully crossed with a workhorse wire (run-through) (Figure 1A), and lesion bed preparation was performed using a 2.5 × 15 mm semi-compliant balloon at the site of the most severe stenosis. However, despite adequate bed preparation, advancing a 3.0 × 38 mm DES proved challenging, with repeated disengagement of the guiding catheter (Figure 1B).

To facilitate stent delivery, a buddy wire was inserted into the distal LAD. During stent positioning, the operator noticed that while the balloon was advancing, the stent appeared to detach from the underlying balloon and was pushed back into the guiding catheter. Suspecting stent detachment (Figure 1C), the operator assessed the situation.

The patient developed acute-onset chest pain, hemodynamic collapse, and ST-segment elevation in the anterior chest leads on electrocardiogram. Given the hemodynamic instability and stent separation, an urgent decision was made to withdraw the entire assembly. However, during retrieval, the stent became dislodged from the assembly and embolized to the bifurcation of the right brachial artery (Figure 1D).

Following this, the patient’s hemodynamics stabilized, with resolution of electrocardiographic changes and relief of chest pain. The clinical dilemma was whether to leave the stent in situ or proceed with its removal. Upon closer fluoroscopic inspection, the stent edges appeared severely deformed, with extrusion of the stent margins. Given the high risk of vessel wall injury and thrombosis, a decision was made to remove the stent.

Multiple attempts were made to pass a guidewire across the dislodged stent for percutaneous retrieval via the same radial access, but these were unsuccessful. An alternative approach via the right femoral artery was then attempted, with multiple attempts to pass coronary wires from the femoral access across the subclavian and into the brachial artery. Despite efforts by multiple interventional experts, the wire could not be advanced across the stent, leading to the failure of both the “two-wire technique” and the “small-balloon technique.”

Given the deformation of the stent, with struts protruding and posing a significant risk, a decision was made to proceed with surgical extraction via arteriotomy rather than adopting the “peripheral parking technique.” The stent was successfully removed through an arteriotomy in the brachial artery, and the patient recovered without complications, being discharged two days later (Figure 1E).

## Discussion

Stent dislodgement during PCI is a rare but potentially fatal complication, with an incidence ranging from 0.32% to 3.4% [2]. Despite the increasing complexity of PCI procedures, the frequency of stent loss has declined over the past two decades due to advancements in stent technology - such as pre-mounted stents, improved cross-sectional profiles, and enhanced delivery systems - and greater operator experience [2,3]. Laarman et al. highlighted that direct stenting carries a higher risk of stent loss due to increased resistance during advancement [4]. Other contributing factors include delivering a stent through a previously deployed stent, stent deformation while navigating a severely stenotic lesion, and deformation caused by the guiding catheter [4]. Additionally, severe coronary calcification and vessel tortuosity further increase the risk [5]. Effective management of a dislodged stent is critical to preventing life-threatening complications, and several available strategies are briefly discussed below.

The “small-balloon technique” involves advancing a balloon across the dislodged stent, inflating it, and carefully withdrawing it into the guide catheter. Alternatively, the stent can be retrieved using a snare or secured against the vessel wall by deploying another stent over it [5]. The “peripheral parking technique” is a novel approach that allows the operator to leave the stent in a peripheral vessel without attempting retrieval [6]. The “two-wire technique” involves advancing a second guidewire distal to the stent, intertwining it with the original guidewire through multiple twists, and then withdrawing both together [7,8].

Specialized instruments such as biliary forceps, Cook retained fragment retrievers, and basket retrieval devices are also available for stent removal. However, these bulky devices are typically reserved for retrieving stents from the aorta or femoral/iliac arteries [8-10]. In contrast, a loop snare, being of smaller caliber, can be used in coronary arteries. In cases where percutaneous retrieval fails, surgical intervention may be necessary, as demonstrated in our case. Cha described instances where vascular surgery was required to retrieve a deformed stent that could not be removed via a radial artery sheath, emphasizing the importance of having surgical backup available [11].

Previously published reports indicate that stent loss can lead to severe complications, including emergency coronary artery bypass grafting (CABG), coronary thrombosis, myocardial infarction, and cerebrovascular embolic events if the stent is lost in the ascending aorta. In some cases, it may even be fatal [11]. A study by Eggebrecht et al. reported a 15% mortality rate among patients with failed stent retrieval, whereas Alfonso et al. found no adverse events among nine patients with dislodged stents [11-13]. Elsner et al. noted that one of six patients required emergency CABG due to coronary ischemia and failed percutaneous stent removal [14].

In our patient, the dislodged stent migrated to the bifurcation of the brachial artery, posing a risk of thrombosis, local ischemia, or vessel wall damage. Preventive measures to reduce such complications include ensuring adequate patient sedation, optimizing lesion preparation before stenting (e.g., pre-dilation, orbital or rotational atherectomy, or shockwave therapy), selecting appropriately sized stents, and ensuring adequate guide catheter support to minimize excessive stent manipulation.

## Conclusions

Stent loss during PCI is uncommon in the modern era but remains more likely in tortuous, heavily calcified, or previously stented coronary arteries. Proper procedural planning, the use of intravascular imaging, and optimized strategies for calcium modification and vessel bed preparation are essential for success. These factors also highlight the importance of high-quality stents with superior radial strength and enhanced trackability across complex lesions.

While most lost stents can be successfully retrieved using various percutaneous techniques, stent loss is still associated with an increased risk of complications, including significant bleeding and the potential need for emergency CABG. Therefore, interventional cardiologists must not only focus on preventing dislodgement but also be highly proficient in retrieval techniques to mitigate serious complications. Additionally, vascular and cardiothoracic surgeons play a critical role in cases where percutaneous retrieval methods fail.
